# Supplementary material for: Encystation stimuli sensing mediated by adenylate cyclase AC2-dependent cAMP signaling in Giardia
Source: Res Sq. 2023 Apr 25:rs.3.rs-2818450. Preprint. [Version 1] doi: 10.21203/rs.3.rs-2818450/v1 (PMC10168462; doi:10.21203/rs.3.rs-2818450/v1)
Supplement: Supplement 1 — SP Fig. 1 cAMP signaling in humans versus Giardia. Diagram depicts canonical cAMP signaling in humans (a) vs cAMP signaling in Giardia. Note that Giardia lacks G-coupled protein receptors and heterotrimeric G-proteins canonically linked to adenylate cyclase regulation. SP Fig.2: Design of GlPKA-NBit. a, GlPKA-NBit is composed of pPKAr::PKAr-LgB and pPKAc::PKAc-SmB, and the control is composed of pPKAr::LgB and pPKAc::PKAc-SmB, b. The absolute luminescence intensity of GlPKA-NBit with or without Glo substrate. C. Relative luminescence intensity of GlPKA-NBit after 0, 0.5, 1, 2, 4 h exposure to encystation medium. The data are from three biological replicates that were normalized to 0h. SP Fig.3: cAMP analogs enhance encystation. a, Intracellular cAMP levels at 0, 0.5, 1, 2, 4 h post encystation. 2×10^6^ cells were collected, lysed, and measured with Caymen cAMP ELISA assay. The absorbance was detected at 405 nm using plate reader, b-c, (b) Western blot and (c) quantification of CWP1 after pretreatment with DMSO, 50µM 8Br-cAMP, and 50µM DB-cAMP. Wild type parasites were pretreated with cAMP analogs for 1h, washed with pre-encystation medium, and exposed to encystation medium. The expression level of CWP1 is normalized to tubulin. d-e, Quantification of cyst viability at 48 h post induction of encystation from parasite with or without 1 h of 8C6P-cAMP pretreatment. (d) Representative images and (e) quantification of viability after 1h pretreatment with 8C6P-cAMP followed by 48h exposure to encystation medium. The water-resistant cysts were stained with fluorescein diacetate (FDA, green=live) and propidium iodine (PI, magenta=dead). Total cysts counted for DMSO-Ctrl n=486, and 8C6P-cAMP n=475. Data are mean ± s.d. Scale bar, 50 µm. SP Fig.4: AC1 is expressed at mid-late stages of encystation. Localization of (a) AC1-mNG, (b) mNG-AC1 (GL50803_14367) at 0, 4, 8, and 16 h exposures to encystation stimuli. c, Relative expression levels of AC1-NLuc after 0, 2, 4, 8, 12, and 16 [file NIHPPrs2818450v1-supplement-1.pdf]

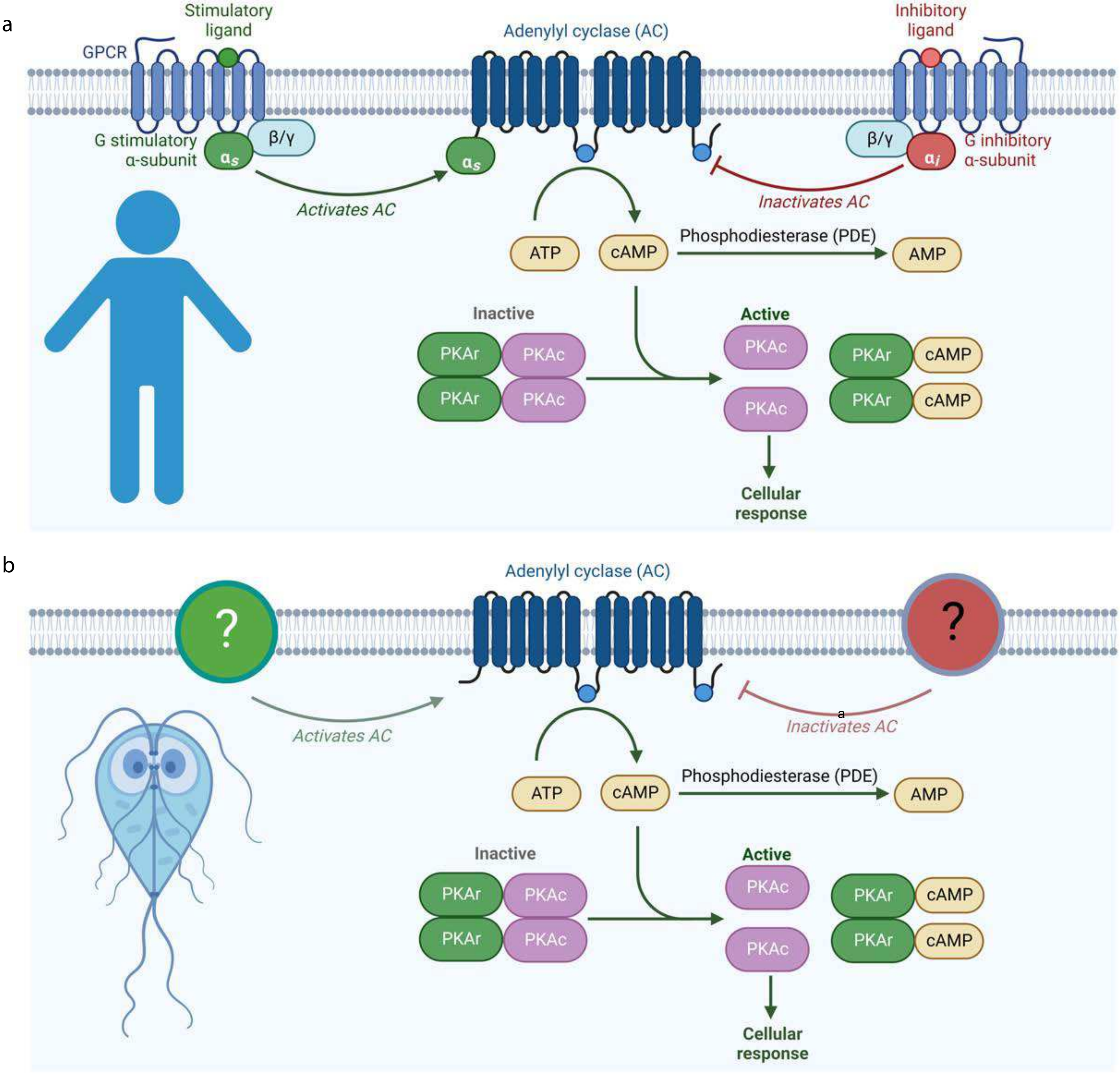

SP Fig. 1 cAMP signaling in humans versus Giardia. Diagram depicts canonical cAMP signaling in humans (a) vs cAMP signaling in Giardia. Note that Giardia lacks G-coupled protein receptors and heterotrimeric G-proteins canonically linked to adenylate cyclase regulation.

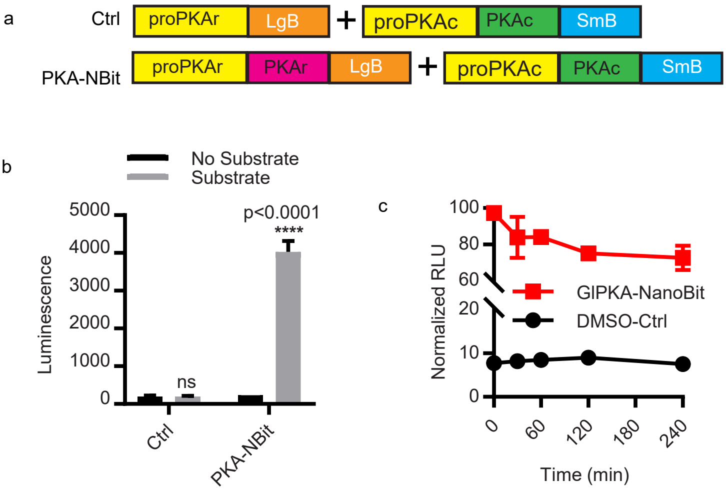

SP Fig.2: Design of GlPKA-NBit. a, GlPKA-NBit is composed of pPKAr::PKAr-LgB and pPKAc::PKAc-SmB, and the control is composed of pPKAr::LgB and pPKAc::PKAc-SmB, b. The absolute luminescence intensity of GlPKA-NBit with or without Glo substrate. C. Relative luminescence intensity of GlPKA-NBit after 0, 0.5, 1, 2, 4 h exposure to encystation medium. The data are from three biological replicates that were normalized to 0h.

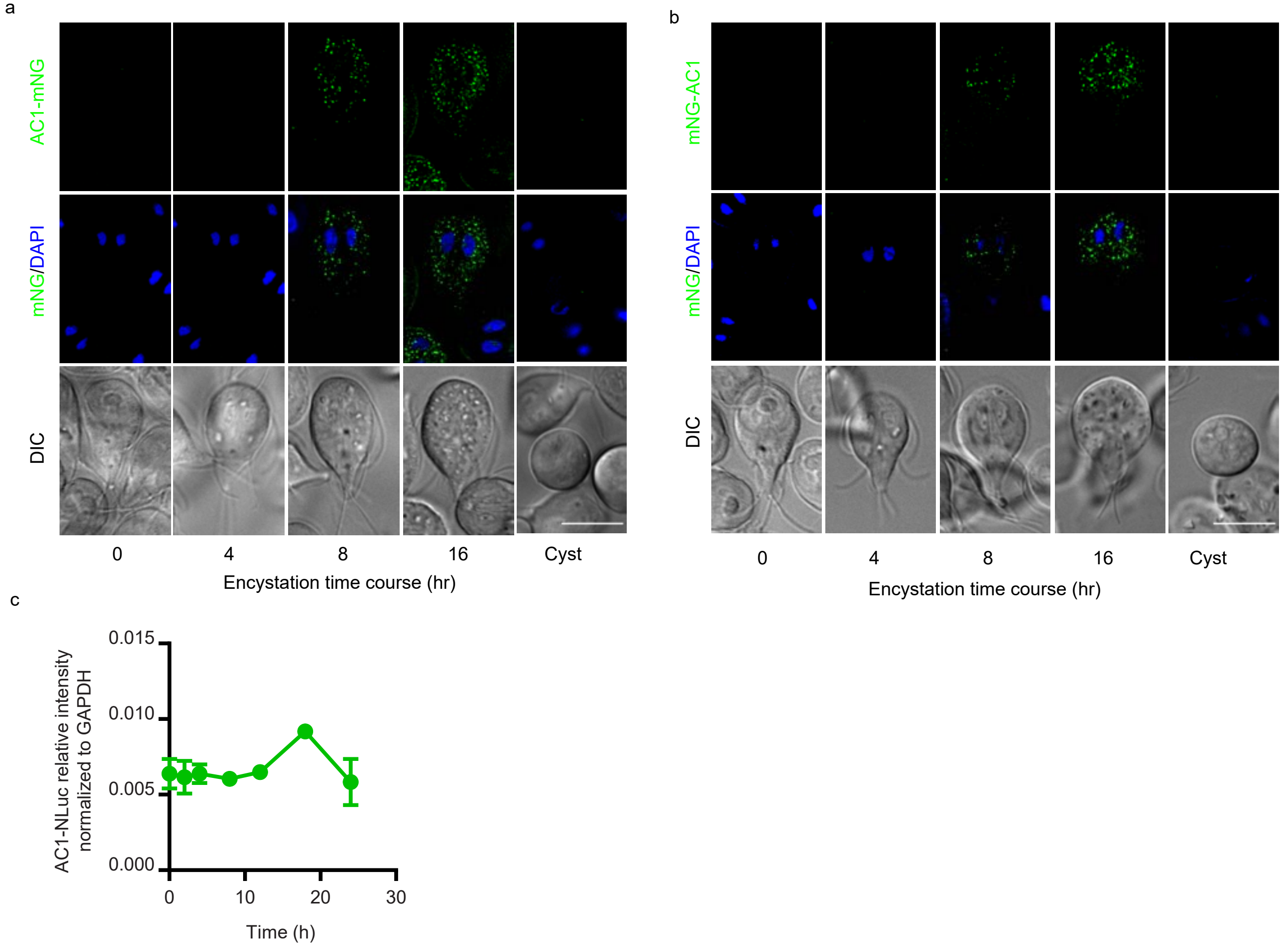

SP Fig.3: cAMP analogs enhance encystation. a, Intracellular cAMP levels at 0, 0.5, 1, 2, 4 h post encystation. 2x106 cells were collected, lysed, and measured with Caymen cAMP ELISA assay. The absorbance was detected at 405 nm using plate reader, b-c, (b) Western blot and (c) quantification of CWP1 after pretreatment with DMSO, 50μM 8Br-cAMP, and 50μM DB-cAMP. Wild type parasites were pretreated with cAMP analogs for 1h, washed with pre-encystation medium, and exposed to encystation medium. The expression level of CWP1 is normalized to tubulin. d-e, Quantification of cyst viability at 48 h post induction of encystation from parasite with or without 1 h of 8C6P-cAMP pretreatment. (d) Representative images and (e) quantification of viability after 1h pretreatment with 8C6P-cAMP followed by 48h exposure to encystation medium. The water-resistant cysts were stained with fluorescein diacetate (FDA, green=live) and propidium iodine (PI, magenta=dead). Total cysts counted for DMSO-Ctrl n=486, and 8C6P-cAMP n=475. Data are mean ± s.d. Scale bar,

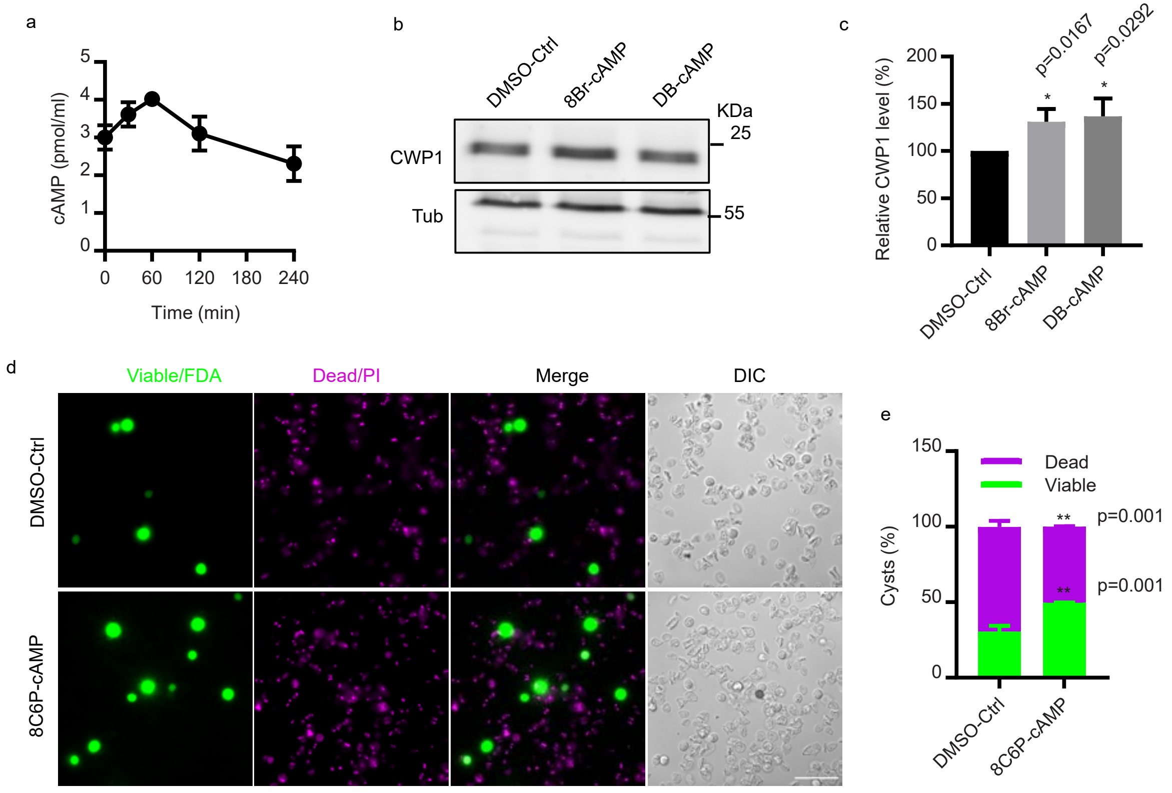

SP Fig.4: AC1 is expressed at mid-late stages of encystation. Localization of (a) AC1-mNG, (b) mNG-AC1 at 0, 4, 8, and 16 h exposures to encystation stimuli. c, Relative expression levels of AC1-NLuc after 0, 2, 4, 8, 12, and 16 h exposures to encystation stimuli. Expression level is normalized to GAPDH::NLuc intensity. Scale bars, 5 µm.

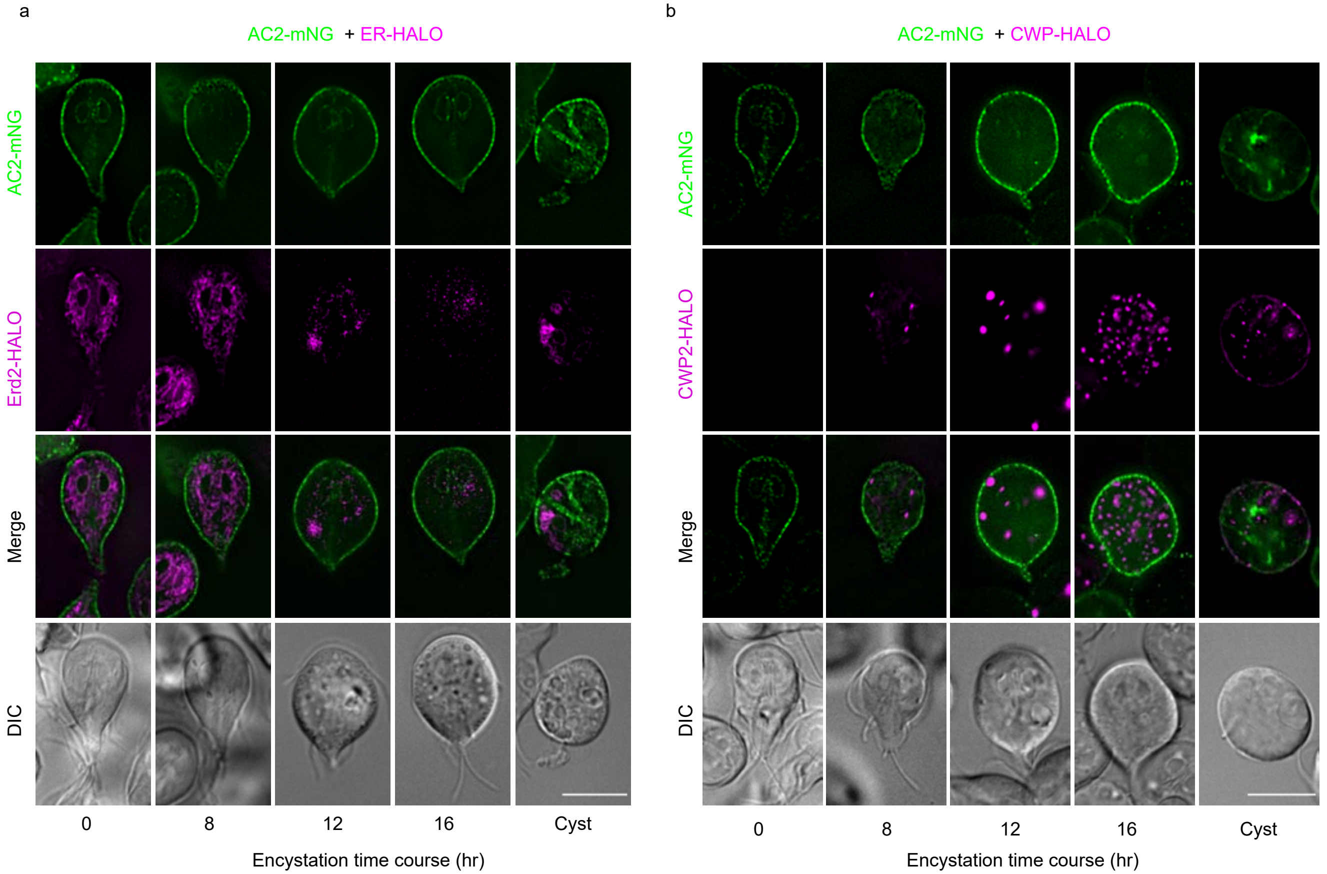

SP Fig.5: Colocalizations of AC2-mNG with ER and ESV marker. a, Colocalization of AC2-mNG and Erd2-Halo (ER marker) at 0, 8, 12, 16, and 24h exposure to encystation medium. b, Colocalization of AC2-mNG and CWP2-Halo (ESV marker) at 0, 8, 12, 16, and 24h exposure to encystation medium. Scale bars, 5 µm.

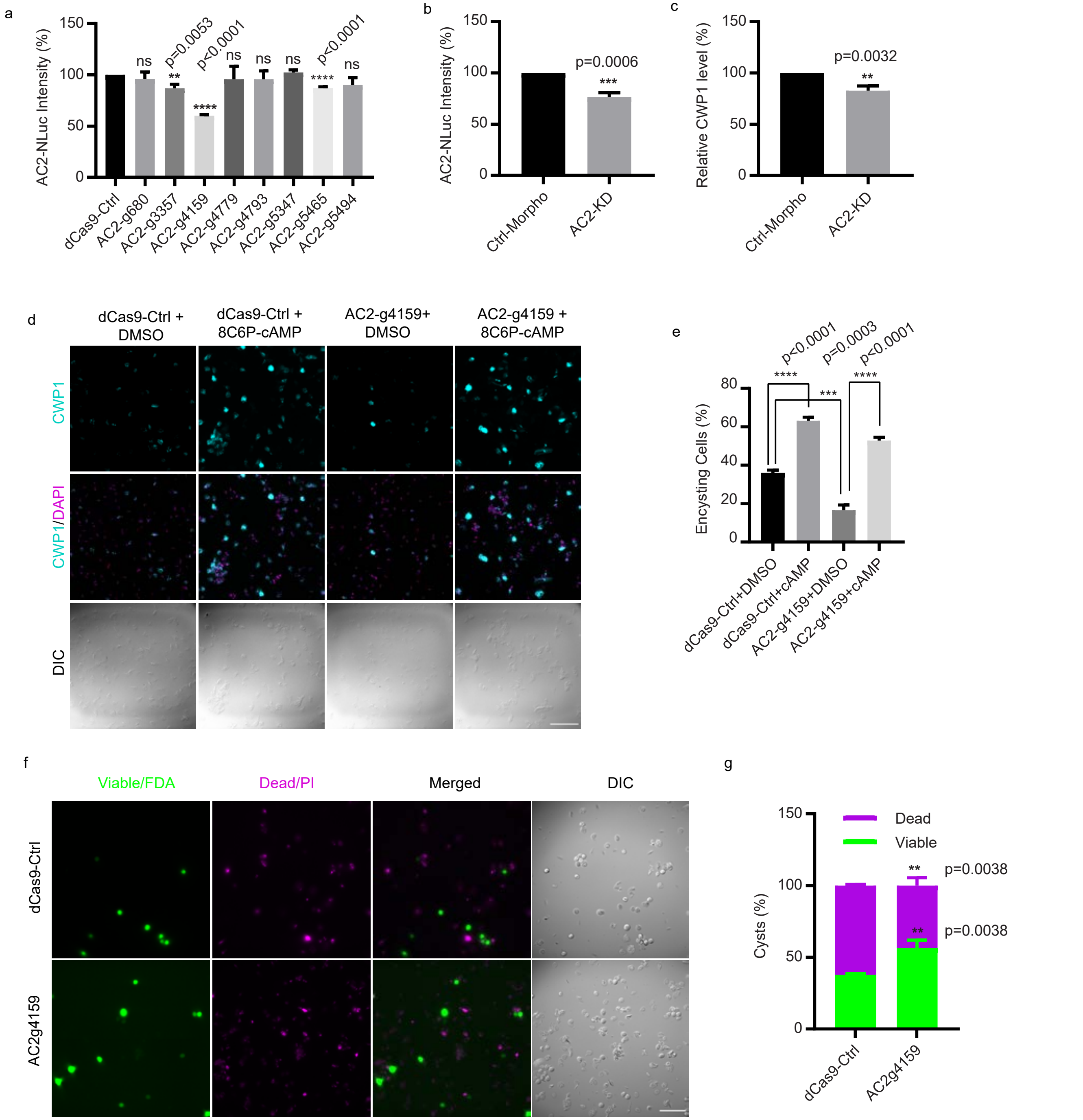

SP Fig.6: Morpholino-mediated AC2 knockdown phenotype is consistent with CRISPRi-mediated AC2 gRNA knock-down. a, Screening of AC2 guide RNAs. Relative AC2-NLuc levels using the indicated CRISPRi gRNAs. b, Relative AC2-NLuc levels using morpholino-mediated AC2 knockdown. c, Relative CWP1-NLuc levels using morpholino-mediated AC2 knockdown.. d-e, Representative images (d) and quantification (e) of 24 h encysting cells from dCas9 control and AC2-g4159 knockdown cell lines with or without 50 μM 8C6P-cAMP. Parasites were pretreated with 50 μM 8C6P-cAMP for 1h followed by 24h exposure to encystation medium (total cells counted for dCas9-Ctrl+DMSO n=1423, dCas9-Ctrl+8C6P-cAMP n=1378, AC2-g4159+DMSO n=1415, and AC2-g4159+8C6P-cAMP n=1283). f-g, Representative images (f) and quantification (g) of dCas9 control and AC2-g4159 derived cysts stained with fluorescein diacetate (FDA, green=live) and propidium iodine (PI, magenta=dead). Data are mean ± s.d. from three biological replicates using student's t-test (cysts counted for dCas9-Ctrl n= 637, and AC2-g4159 n=571). Scale bars, 50 μm.

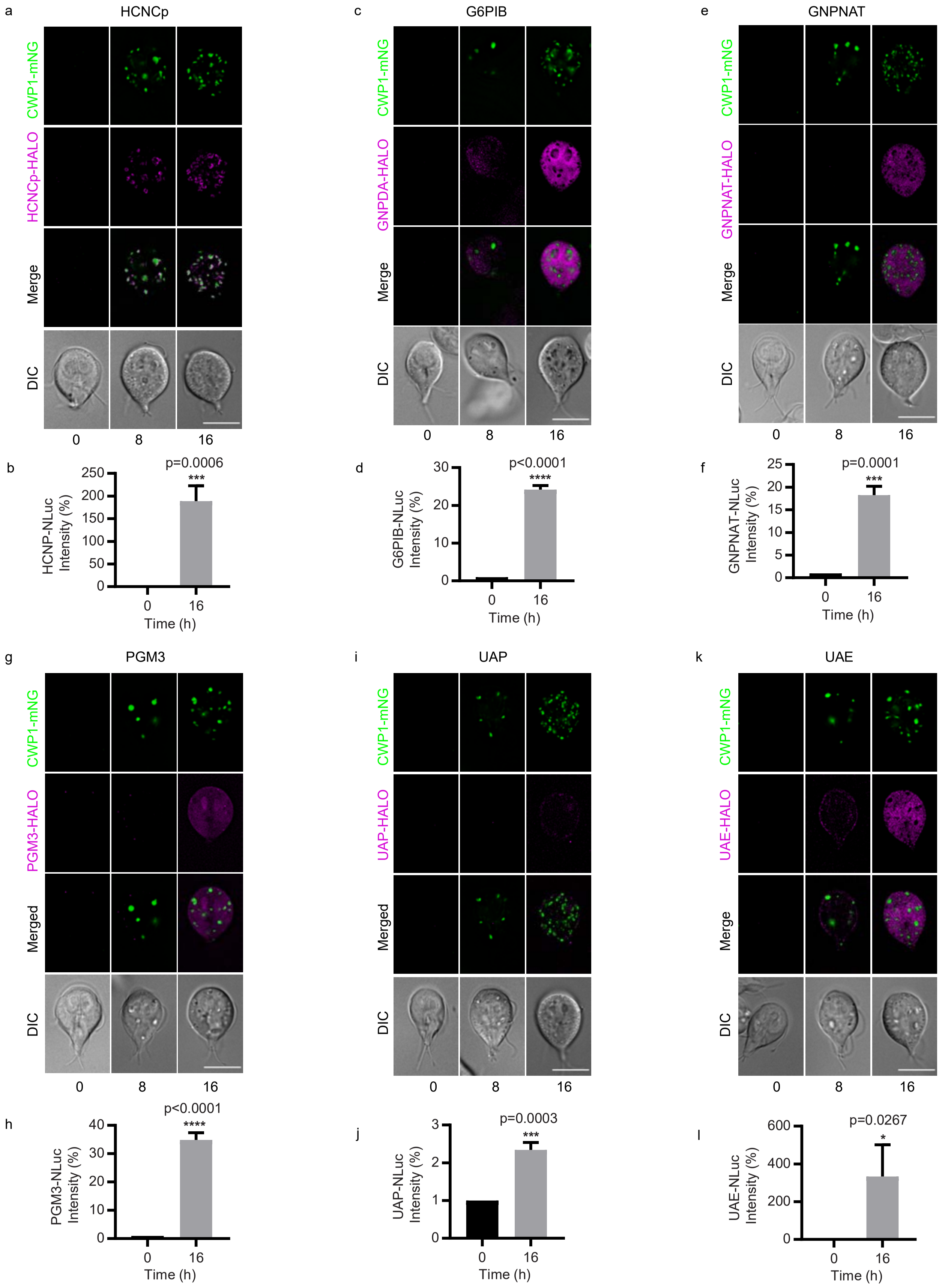
a
HCNCp
CWP1-mNG
HCNCp-HALO
Merge
DIC
0
8
16
b
HCNP-NLuc
Intensity (%)
p=0.0006
***
Time (h)
c
G6PIB
CWP1-mNG
GNPDA-HALO
Merge
DIC
d
G6PIB-NLuc
Intensity (%)
p<0.0001
****
Time (h)
e
GNPNAT
CWP1-mNG
GNPNAT-HALO
Merge
DIC
f
GNPNAT-NLuc
Intensity (%)
p=0.0001
***
Time (h)
g
PGM3
CWP1-mNG
PGM3-HALO
Merged
DIC
h
PGM3-NLuc
Intensity (%)
p<0.0001
****
Time (h)
i
UAP
CWP1-mNG
UAP-HALO
Merged
DIC
j
UAP-NLuc
Intensity (%)
p=0.0003
***
Time (h)
k
UAE
CWP1-mNG
UAE-HALO
Merge
DIC
l
UAE-NLuc
Intensity (%)
p=0.0267
*
Time (h)

SP Fig.7: GalNAc biosynthesis enzymes are upregulated at 16h post induction of encystation. ***a-c***, Relative expression levels of HCNCp and GalNAc biosynthesis enzymes for (***a,b***) HCNCp-Halo and HCNCp-NLuc (GL50803_40376), (***c,d***) G6PI-B-Halo and G6PI-B-NLuc (GL50803_8245), (***e,f***) GNPNAT-Halo and GNPNAT-NLuc (GL50803_14259), (***g,h***) PGM3-Halo and PGM3-NLuc (GL50803_16069), (***i-j***) UAP-Halo and UAP-NLuc (GL50803_16217), (***k,l***) UAE-Halo and UAE-NLuc (GL50803_7982). HCNCp and GalNAc biosynthesis enzymes were tagged with Halo and labeled with Halo tag ligand JF646. CWP1 was tagged with mNeonGreen. Halo tagged cell lines were imaged at 0, 8, 16 h post exposure to encystation medium. All images in were taken with equal exposure. NLuc-tagged cell lines were measured at 16 h post induction of encystation using plate reader. Data are mean ± s.d. from three biological replicates using student's t-test. Scale bars, 5 μm.

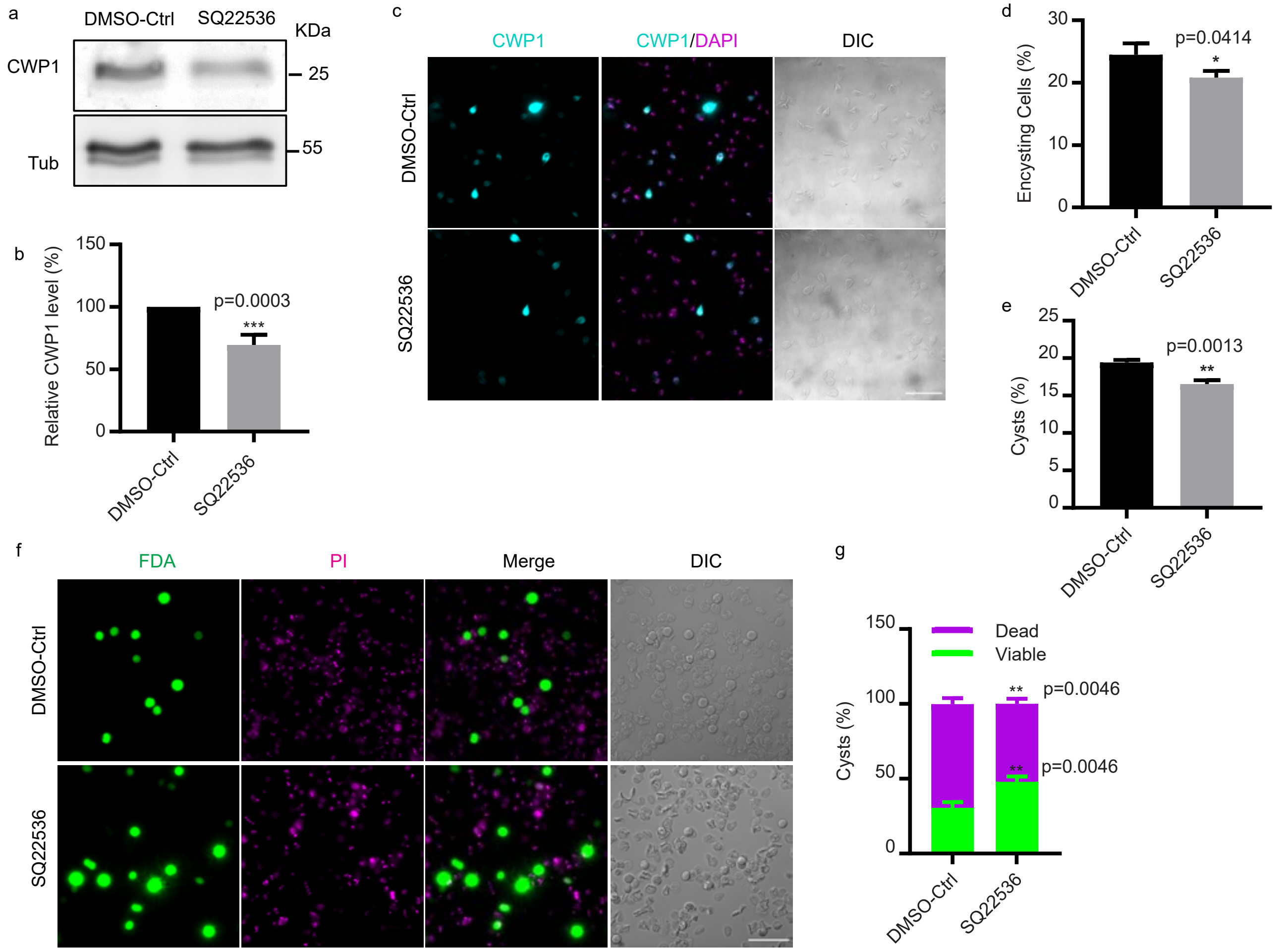

SP Fig.8: AC inhibitor SQ22536 impairs encystation. a-b, Western blot (a) and quantification (b) of CWP1 and tubulin from 1 h pretreatment of DMSO control and SQ22536 at 4h post exposure to encystation medium. c-d, Representative images (c) and quantification (d) of 24 h encysting cells from DMSO control and SQ22536 treated parasite. Parasites were pretreated with 10 µM SQ22536 for 1h followed by 24h exposure to encystation medium (total cells counted for DMSO-Ctrl n= 1002, and SQ22536 n=1028). e, Quantification of mature cysts at 48 h post induction of encystation from parasites pretreated with DMSO or SQ22536 for 1 h. Cyst counts were performed by hemocytometer (total cells counted for DMSO-Ctrl n=1730, and SQ22536 n=1463). Data are mean ± s.d. f-g, Representative images (f) and quantification (g) of DMSO control and SQ22536-treated cysts stained with fluorescein diacetate (FDA, green=live) and propidium iodine (PI, magenta=dead). Data are mean ± s.d. from three biological replicates using student's t-test (cysts counted for dCas9-ctrl n=486, and SQ22536 n=441). Scale bars, 50 µm.

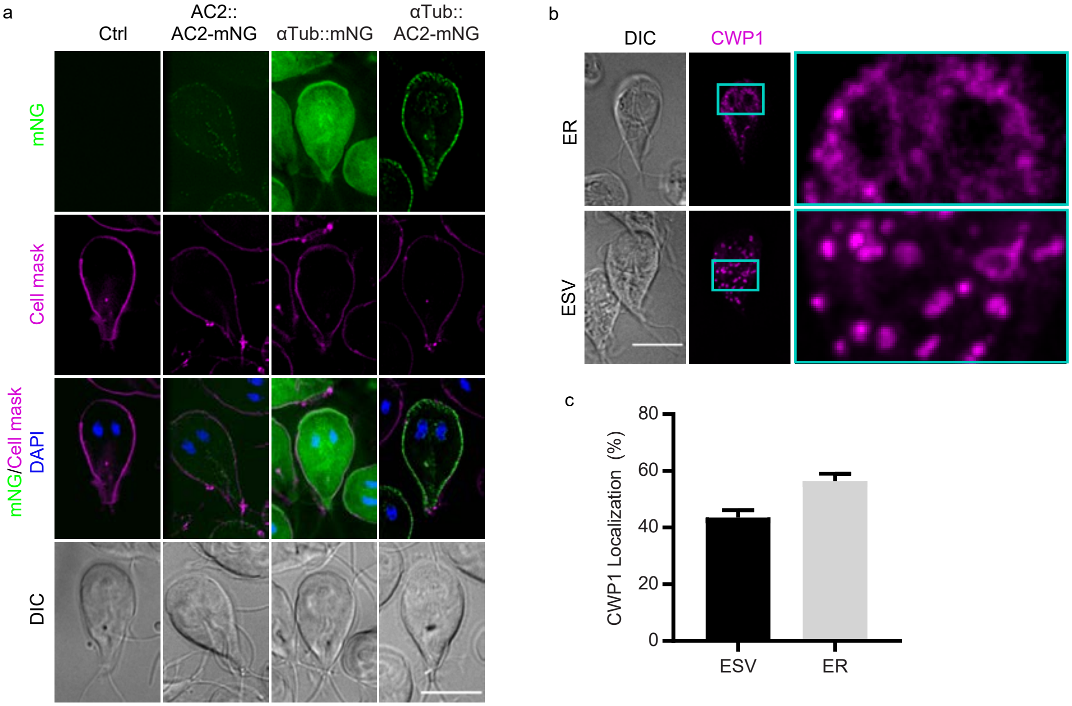

SP Fig.9: AC2 overexpression initiates encystation. a, Representative images of mNeonGreen from the indicated cell lines. The images were taken using equal exposures. b-c, Representative IFA images (b) and quantification (c) of encysting cells with ER or ESV localized CWP1. Data are mean ± s.d. from three biological replicates using student's t-test (total cell counted n=239). Scale bars, 5 μm.
